# A splicing mutation of the *FLCN* gene is associated with Birt-Hogg-Dubé syndrome characterized by familial and recurrent spontaneous pneumothorax: A case report

**DOI:** 10.1097/MD.0000000000034241

**Published:** 2023-07-07

**Authors:** Hua Xiao, Feng Chi, Shuai Li, Tao Wang, Bin Bai, Jia Hou, Xiahui Ge

**Affiliations:** a Department of Respiratory Medicine, Seventh People’s Hospital of Shanghai University of Traditional Chinese Medicine, Shanghai, P.R. China; b Department of Imaging and Nuclear Medicine, The First Affiliated Hospital of Zhengzhou University, Zhengzhou, P.R. China; c Key Laboratory of Digital Technology in Medical Diagnostics of Zhejiang Province, Dian Diagnostics Group CO., Ltd., Hangzhou, Zhejiang Province, P.R. China; d Department of Gastrointestinal Surgery, Shanghai Baoshan Hospital of Integrated Traditional Chinese and Western Medicine, Shanghai, P.R. China; e Department of Respiratory and Critical Care Medicine, General Hospital of Ningxia Medical University, Ningxia, P.R. China; f Department of Respiratory and Critical Care Medicine, Shanghai Ninth People’s Hospital, Shanghai Jiao Tong University School of Medicine, Shanghai, P.R. China.

**Keywords:** Birt-Hogg-Dubé syndrome, *FLCN* gene, recurrent spontaneous pneumothorax, WES

## Abstract

Birt-Hogg-Dubé (BHD) syndrome is a rare autosomal recessive genetic disorder caused mainly by mutations in the tumor suppressor *FLCN* gene. Tumors caused by *FLCN* mutations are frequently benign and develop in skin, lungs, kidney, and other organs, leading to a variety of phenotypes that make early diagnoses of BHD challenging.

**Patient concerns:**

A 51-year-old female was admitted to Shanghai Seventh People Hospital due to chest congestion and dyspnea that had persisted for 3 years and aggravated for 1 month. She had been diagnosed with pneumothorax prior to this submission, but the etiology was unknown.

**Diagnoses:**

Chest computed tomography (CT) revealed multiple pulmonary cysts and pneumothorax, and her family members shared similar manifestation. Whole-exome sequencing analysis indicated a heterozygous *FLCN* splicing mutation (c.1432 + 1G > A; rs755959303), which was a pathogenic variant indicated in ClinVar. Based on *FLCN* mutation and the family history of pulmonary cysts and pneumothorax, BHD syndrome was finally diagnosed, which had been delayed for 3 years since her first pneumothorax.

**Interventions:**

Pulmonary bullectomy and pleurodesis were finally conducted due to the poor effects of thoracic close drainage.

**Outcomes:**

Her pneumothorax was resolved, and no recurrence was found in 2 years.

**Lessons:**

Our study highlights the importance of genetic analysis in diagnosis and clinical management of BHD syndrome.

## 1. Introduction

The human *FLCN* gene on chromosome 17p11.2 contains 14 exons and encodes a highly conserved protein called folliculin, which is a classic tumor suppressor involved in the regulation of cell growth and proliferation.^[1,2]^ Growing evidence has shown that mutations of the *FLCN* gene mainly contributes to the pathogenesis of Birt-Hogg-Dubé (BHD) syndrome (OMIM: 135150), an autosomal recessive disease characterized by benign tumors in skin, lungs, kidney, and other organs.^[1–5]^ The location, onset, and progression of benign tumors depend on age, and may have racial/ethnic differences.^[3,6–8]^ Such a heterogeneity makes early diagnoses of BHD syndrome challenging and further increases the risk of benign tumors becoming malignant.^[3,7–11]^

Pulmonary cyst is one of the main clinical manifestations of BHD syndrome and more commonly seen in Asian BHD syndrome patients than Caucasians, with an incidence of over 90% reported in Chinese and Japanese.^[3,6,8,12]^ Young BHD syndrome patients with pulmonary cysts may not have obvious symptoms, but when they reach the middle age, pulmonary cysts tend to rupture, resulting in spontaneous pneumothorax with high recurrence rate.^[3,13,14]^ In general, BHD syndrome patients with pneumothorax always complain of chest pain, dyspnea, cough and rapid breathing and heartbeat, and chest images can easily help to find pneumothorax.^[3,13,15]^ However, none of these characteristics is specific to BHD syndrome, and patients with pneumothorax can easily be missed or delayed for BHD syndrome diagnosis, even for over a decade.^[7,14]^

Here, we report a 51-year-old female patient with recurrent spontaneous pneumothorax (3 episodes) that caused by multiple pulmonary cysts, and with a family history of pulmonary cysts and pneumothorax. Meanwhile, cystic changes in her kidney and liver were also detected. We identified a heterozygous splicing mutation in the *FLCN* gene (c.1432 + 1G > A; rs755959303) by whole-exome sequencing analysis and made the final diagnosis of BHD syndrome, which had been delayed for 3 years since her first pneumothorax. Our study highlights the importance of genetic analysis in diagnosis and clinical management of BHD syndrome.

## 2. Case description

### 2.1. Medical history and family history

**Figure 1.**
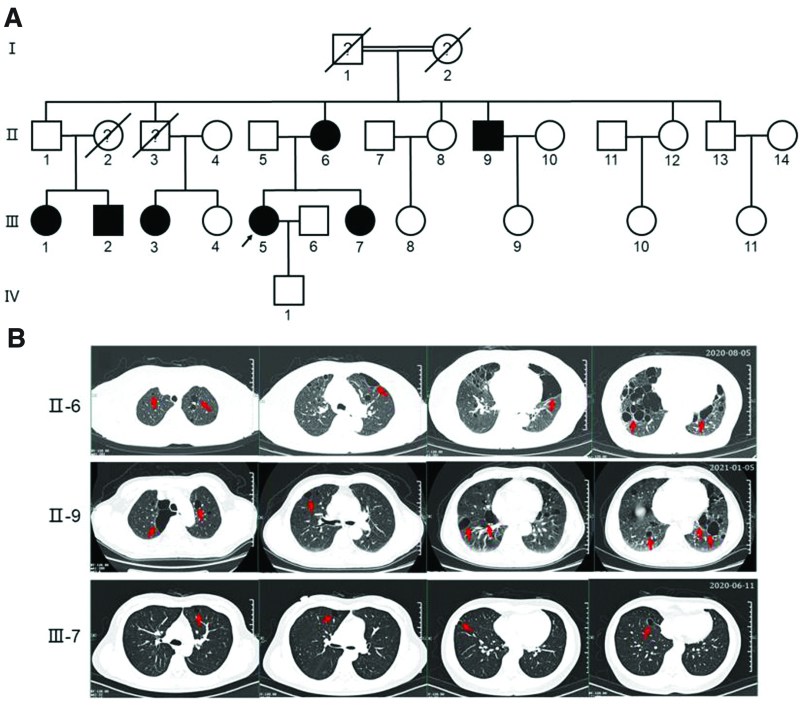
Pedigree analysis and high-resolution chest CT images obtained from the patient family. (A) The patient, that is, the proband (III-5), enrolled in this study is indicated with an arrow. The proband’s grandparents (I-1, I-2) are consanguineous as indicated with a double horizontal line. Family members who are affected by pulmonary cysts are indicated with solid black. Deceased family members are indicated with diagonal line. Question mark indicates an unknown history of pulmonary cysts or other lung diseases. (B) Representative chest cross section CT images from the family members with pulmonary cysts (red arrows). Thin-walled pulmonary cysts with uneven size and form are scattered throughout the lungs bilaterally and asymmetrically. Compared to II-6 and II-9, both the size and number of pulmonary cysts in III-7 are relatively smaller. CT images was obtained from regular physical checkup in Shanghai Seventh People Hospital. Recording dates are indicated. CT = computed tomography.

A 51-year-old female patient (III-5; Fig. 1A) was admitted to Shanghai Seventh People Hospital in September 2021 with chest congestion and dyspnea that had persisted for 3 years and aggravated for 1 month. The past medical history showed that she had 2 previous admissions to other hospitals due to similar respiratory symptoms. During her first hospitalization in 2018, she had been diagnosed with spontaneous pneumothorax. Lymphangioleiomyomatosis was excluded at that time since serum VEGF-D was 319.79 pg/mL. She received thoracic close drainage, and her symptoms including out of breath and chest pain alleviated and was discharged. One month before visiting us, her chest congestion and dyspnea recurred without obvious predisposing cause, followed by hospital re-admission and thoracic catheterization for 1 week. Shortly after withdrawal of the tube, the pneumothorax recurred. In order to seek further diagnosis and treatment, she was transferred to our hospital.

Meanwhile, we were also informed that she had a family history of pulmonary cysts and pneumothorax, and her grandparents were consanguineous (Fig. 1A). Her mother, her younger sister, 1 of her uncles, and 3 of her cousins all suffered from pulmonary cysts with varying severity (Fig. 1A and B). Except the patient’s younger sister, all affected family members had experienced at least 1 episode of spontaneous pneumothorax (Table 1). Other hereditary disease history and smoking history were denied by the patient.

**Table 1:** The summary of the family members who are affected by pulmonary cysts.

Family members	Age	Pulmonary cysts yes/no	Pneumothorax		
			Yes/no	Onset age	Episode(s)
II-6	75 yr	Yes	Yes	50 yr	2
II-9	67 yr	Yes	Yes	50 yr	1
III-1	Unknown	Yes	Yes	Unknown	
III-2	Unknown	Yes	Yes	Unknown	
III-3	Unknown	Yes	Yes	Unknown	
III-5	51 yr	Yes	Yes	48 yr	3
III-7	47 yr	Yes	No	No	

### 2.2. Clinical examination and management

**Figure 2.**
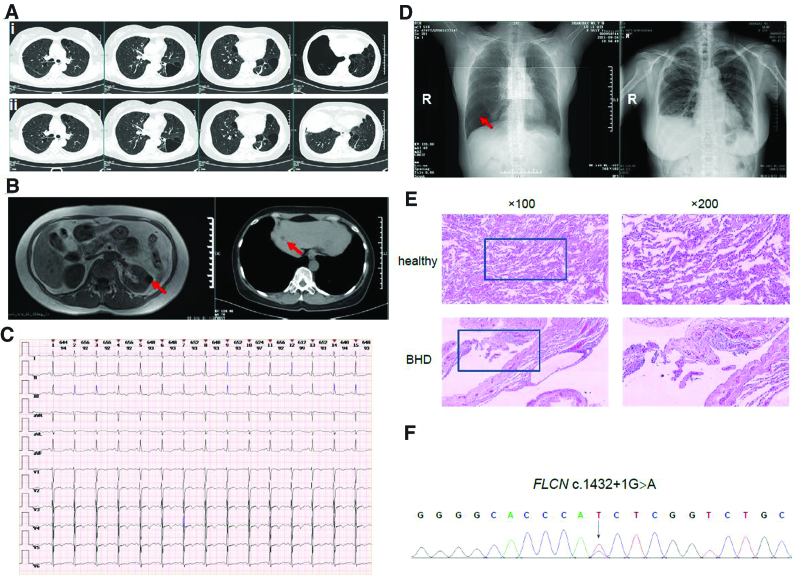
The imaging, ECG, pathological, and genetic findings obtained from the patient. (A) Representative chest cross section CT images on admission (i; upper panels) and after pulmonary bullectomy and pleurodesis (ii; lower panels). (B) CT images show renal cyst (left) and liver cyst (right), which are indicated with red arrows. (C) On admission, the 12-lead ECG shows ST-segment horizontal depression (0.5–1.0 mV) in limb leads (I–III, aVF); reciprocal ST-segment elevation (0.5 mV) in the leads of aVL, aVR, and V2-V6; T-wave inversion in the II, III, aVF, and V4-V6 leads. (D) On admission, CT scout view (left) shows right-side pneumothorax that indicated with red arrow; after pulmonary bullectomy and pleurodesis, chest X-ray (right) shows the fully expanded right lung. (E) Histology following H&E stain (upper panels, healthy lung tissues; lower panels, surgical lung lesions from the patient; magnification times are indicated). Histological results show the dilated alveoli and cysts, vascular congestion and hemangiectasis within the cysts, and lymphocytes infiltration in the lung lesions from the patient. Alveoli are presented in uniform morphology in healthy lung. Enlarged area is circled by a box. (F) Sanger sequencing confirms the heterozygous *FLCN* c.1432 + 1G > A variant in the patient. CT = computed tomography, ECG = electrocardiogram.

On admission, physical examination revealed a normal body temperature (36.5°C), a blood pressure of 151/102 mm Hg (normal range, <135/85 mm Hg), a heart rate of 120 beats/min (normal range, 60–100 beats/min), a breathing rate of 22 breaths/min (normal range, 12–20 breaths/min), and an oxygen saturation of 90% on room air (normal range, >94%). Respiratory movement of both side was natural and symmetrical, and thoracic deformity was not observed. A decreased tactile fremitus at right chest was revealed by palpation. Lung percussion further indicated a drum-like sound of the right side and auscultation revealed a decreased breathing sound at the same region, while pulmonary rale or crackles and cardiac murmur were not heard. High-resolution chest computed tomography (CT) showed bilateral multiple thin-walled pulmonary cysts and the right side lung parenchyma was significantly compressed (~75%) by right side pneumothorax, while displacement of trachea and mediastinum was not obvious (Fig. 2A). Furthermore, in her upper left kidney and right lobe of liver, small nodules were also found in chest CT images (Fig. 2B). Abdominal ultrasound confirmed the presence of typical anechoic and well-delimited cystic lesions in the left kidney (largest cyst size: 1.8 × 1.5 cm) and in the liver (largest cyst size: 1.2 × 1.1 cm), while no other abdominal abnormality was found. Moreover, 12-leads regular electrocardiogram further revealed characteristics of myocardial ischemia (Fig. 2C). There were no abnormalities regarding her neurological and musculoskeletal examination. It is worthy to note that we also observed miliary papules on the skin of her face and neck, but pathological examination of skin biopsy tissues showed no abnormality.

On the first day of her admission, oxygen was immediately given and thoracic catheterization was conducted to drain air from the chest. However, 2 weeks later, a persistent leak of air was still observed in the water-sealed bottle. Thoracic surgeon was then consulted, and right-sided video-assisted thoracoscopic bullectomy and pleurodesis were performed on the patient. Pathological analysis of the surgical lesions demonstrated pulmonary cysts and bullae formation with focal vascular congestion (Fig. 2D). Postoperative chest images showed that the right lung was fully expanded (Fig. 2A and E), and her chest congestion and dyspnea was also resolved. One week after surgery, the patient was discharged with excellent recovery, and a regular surveillance of cysts in lung, kidney, and liver was strongly recommended.

### 2.3. Genetic testing and final diagnosis

In order to determine the etiology of recurrent pneumothorax, we also obtained the patient written informed consent and further collected her peripheral blood samples for whole-exome sequencing on Illumina NovaSeq 6000 platform. Following the methods provided by the manufacturers, genomic DNA was extracted using the QIAamp DNA Blood Midi KIT (Qiagen, Dusseldorf, Germany), and the exome sequences were enriched using the IDT xGen Exome Research Panel v1.0 (Integrated DNA Technologies, Coralville, IA). Via standard bioinformatics analysis and variant annotation (reference human genome GRCh37/hg19),^[16]^ we determined that the recurrent pneumothorax is most likely caused by a heterozygous splicing mutation in the *FLCN* gene (c.1432 + 1G > A; rs755959303), which was further confirmed by Sanger sequencing (Fig. 2E). This variant occurs at a canonical splice site and results in a G to A transition at 1 nucleotide in intron 12, thus *FLCN* c.1432 + 1G > A is expected to be deleterious by producing aberrant RNA splicing and/or protein products. Importantly, *FLCN* c.1432 + 1G > A has been reported in patients with BHD syndrome^[17,18]^ and colorectal cancer,^[19]^ whereas it has not been recorded in any general population databases. According to the American College of Medical Genetics guidelines, *FLCN* c.1432 + 1G > A is classified as likely pathogenic or pathogenic in ClinVar database (https://www.ncbi.nlm.nih.gov/clinvar/variation/VCV000253252.16). On the basis of clinical and genetic evidence, we concluded that *FLCN* c.1432 + 1G > A contributes to the pathogenesis of pulmonary cysts and recurrent pneumothorax in the patient and confirmed the final diagnosis of BHD syndrome, which had been delayed for 3 years since her first episode of pneumothorax. Unfortunately, other family members declined genetic testing because of the potential psychological burden.

## 3. Discussion

BHD syndrome is a rare and highly heterogeneous disease, and many cases may be undiagnosed or misdiagnosed. The exact prevalence of BHD syndrome is still unknown, with only a few hundred BHD syndrome families with *FLCN* mutations reported so far and initial symptom(s) vary with each individual.^[3]^ For example, a triad of skin lesions, that is, fibrofolliculomas, trichodiscomas, and acrochordons, are the symptoms first identified in BHD syndrome patients and has long been recognized as the hallmark of BHD syndrome.^[1,3,5]^ In fact, many studies have shown that the prevalence of skin lesions in Asian BHD syndrome patients is less than half, even if skin lesions occur, it does not cause particular concern to patients or clinicians.^[6,8,12]^ In this study, we did observe skin changes in the patient, which yet were histologically normal. Nevertheless, we still can not rule out the potential influence of the sampling sites on the final pathology results. Therefore, it is difficult to recognize suspicious BHD syndrome patients early if skin changes alone are relied upon, especially for non-dermatologists.

Compared to skin lesions, pulmonary cysts are found to be more frequent in Asian BHD syndrome patients.^[6,8,12]^ BHD syndrome-related pulmonary cysts are usually thin-walled, irregular-shaped and unevenly distributed in both lungs, especially in the lower lobe and near the mediastinum.^[15]^ Pulmonary cysts may not cause noticeable respiratory symptoms until cysts rupture and pneumothorax happen at the advanced stage. The average onset age of pneumothorax also varies greatly among BHD syndrome patients, generally appear in the age of 30s or 40s,^[3,14]^ which is relatively earlier than the pneumothorax onset age of our patient and her relatives. It has also been shown that female BHD syndrome patients tend to had younger onset age of pneumothorax than male,^[13]^ however, this view remains controversial.^[3,20]^ Another distinct feature of BHD syndrome-related pneumothorax is the tendency to relapse. In large cohort studies, about 80% of BHD patients experience 3 episodes of pneumothorax on average.^[14,20]^ Recurrent pneumothorax can lead to rare but potentially life-threatening complications such as respiratory distress, cardiac arrest, shock, and multiple organ dysfunction. In addition, cystic tumors may also affect the kidney, liver, and other organs, and possess a high risk of transforming into malignant ones.^[3,7,10]^ Thus, it is critical to be aware of the correlation between recurrent pneumothorax and BHD syndrome and to consider comprehensive examination and early intervention, even if patients only have seemingly ordinary respiratory symptoms.

The definitive diagnosis of BHD syndrome heavily relies on a genetic evaluation of the major BHD syndrome-causing gene, that is, *FLCN* gene. HGMD database has included more than 200 pathogenic or likely pathogenic *FLCN* variants. Most *FLCN* variants are truncating variants, such as splice site, frameshift, and nonsense variants. As for *FLCN* c.1432 + 1G > A, also known as IVS12 + 1G > A, independent studies have discovered this classical splicing site variant in BHD syndrome patients^[17,18]^; our study is the first to report its occurrence in Asian populations. More recently, Melanie Vog et al, identified a different base exchange at the same nucleotide site of *FLCN* gene, that is, c.1432 + 1G > T, in an Australian BHD syndrome patient.^[21]^ This finding further strengthen the association between this splice site and BHD syndrome pathogenicity. Thus, we conclude that the *FLCN* c.1432 + 1G > A is the major pathogenic cause of the recurrent pneumothorax in our patient, and confirm the final diagnosis of BHD syndrome.

In this study, to explore the effect *FLCN* c.1432 + 1G > A variant at protein level, we also applied immunohistochemistry analysis and the results showed that the heterozygous *FLCN* c.1432 + 1G > A variant has a negligible effect on total folliculin protein expression level in the surgical lung lesions. In consistent with the low cell type specificity of folliculin expression,^[22]^ we found that folliculin is widely expressed in bronchiolar epithelial cells, pneumocytes, and stromal cells in healthy lung tissues (Supplementary Fig. 1, http://links.lww.com/MD/J238). There is no significant change in the cell types expressing folliculin in the lung lesions of our patient, yet folliculin is strongly positively expressed in pneumocytes surrounding the cystic wall (Supplementary Fig. 1, http://links.lww.com/MD/J238). Future investigation is still required to determine the exact pathogenic effect and underlying mechanism of *FLCN* c.1432 + 1G > A variant on lung cell function.

In terms of the treatment of BHD syndrome-related pneumothorax, there is still no universally accepted consensus. Many studies have proven that pulmonary bullectomy and pleurodesis offer a better outcome in controlling the recurrence rate compared to routine conservative management.^[6,20,23]^ Liu Y.G., et al, recently reported that the recurrence rate of BHD syndrome-related pneumothorax was 9.1% and 53.1% following surgery and conservative therapy, respectively, but the information of the follow-up period was missing.^[23]^ Thus, there is still a need for extensive research into the long-term therapeutic effects of surgery on BHD syndrome-related pneumothorax. In this investigated family, both the patient and her mother received pulmonary bullectomy and pleurodesis, and until the time of writing, no recurrence of pneumothorax was found in two and ten years, respectively. Therefore, early diagnosis of BHD syndrome in suspected patients with recurrent pneumothorax also plays an important role in making timely decision regarding surgery.

## 4. Conclusions

In summary, we report a patient with familial and recurrent spontaneous pneumothorax. With the identification of *FLCN* c.1432 + 1G > A variant, we further reveal the etiology of recurrent pneumothorax, and confirm the diagnosis of BHD syndrome. Instead of conservative therapy, her recurrent pneumothorax was resolved by pulmonary bullectomy and pleurodesis. Our study highlights the importance of genetic analysis in diagnosis and clinical management of BHD syndrome.

## Acknowledgments

The authors thank the patient for participating in this study and the medical staff involved in the patient diagnosis and treatment.

## Author contributions

**Conceptualization:** Feng Chi.

**Data curation:** Feng Chi.

**Funding acquisition:** Xiahui Ge.

**Investigation:** Shuai Li, Bin Bai.

**Project administration:** Hua Xiao, Feng Chi, Shuai Li.

**Supervision:** Jia Hou, Xiahui Ge.

**Visualization:** Bin Bai.

**Writing – original draft:** Hua Xiao.

**Writing – review & editing:** Tao Wang, Jia Hou, Xiahui Ge.

## Supplementary Material


