# Supplementary material for: A splicing mutation of the FLCN gene is associated with Birt-Hogg-Dubé syndrome characterized by familial and recurrent spontaneous pneumothorax: A case report
Source: Medicine (Baltimore). 2023 Jul 7;102(27):e34241. doi: 10.1097/MD.0000000000034241 (PMC10328714; doi:10.1097/MD.0000000000034241)

Supplementary Figure 1. Immunohistochemistry staining analysis the expression of folliculin in the healthy lung tissues (A) and the surgical lung lesions of the patient (B). Rabbit anti-folliculin polyclonal antibody (abs111703; 1:500 dilution) was purchased from Absin Bioscience.

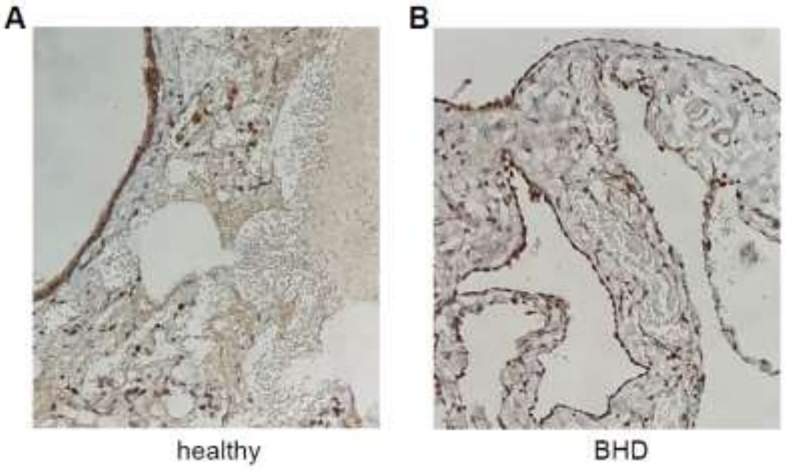

Supplement: Supplementary file 1 [file medi-102-e34241-s001.pdf]
